# Case Report: Zonisamide-induced DRESS syndrome with progression toward SJS: the first Chinese case and review of the literature on ZNS-associated SCARs

**DOI:** 10.3389/fmed.2025.1708376

**Published:** 2025-11-12

**Authors:** Juan Wen, Kang Du, Difang Shi, Yue Wang, Ruohong Xue, Baogang Huang, Xi Liang, Huijuan Fan, Fengming Xu, Haohao Wu

**Affiliations:** 1 Department of Neurology, Yunnan Qujing Central Hospital (Qujing First People’s Hospital), Yunnan, Qujing, China; 2 Department of Neurology, Guizhou Panjiang Investment Holding (Group) General Hospital, Liupanshui, China; 3 Department of Thoracic Surgery I, The Third Affiliated Hospital of Kunming Medical University, Yunnan Cancer Hospital, Peking University Cancer Hospital Yunnan, Kunming, China; 4 Department of Paediatrics, Qujing First People's Hospital, Qujing, China; 5 Department of the First Neurology, The First Affiliated Hospital of Kunming Medical University, Kunming, China

**Keywords:** ZNS, DRESS, SJS, TEN, SCARs

## Abstract

Zonisamide (ZNS), a sulfonamide-derived anticonvulsant, is increasingly used for epilepsy but remains an underrecognized cause of severe cutaneous adverse reactions (SCARs). This study reports the first documented case in China of ZNS-induced drug rash with eosinophilia and systemic symptoms (DRESS), which initially presented as painful papules and herpetiform lesions on the lumbar, abdominal, and facial regions. The patient also exhibited a potential risk of progression to Stevens-Johnson syndrome (SJS) in the context of acute liver failure. Notably, the patient’s latency period was 57 days, representing the second-longest interval reported in the literature. Furthermore, we provide a comprehensive review of ZNS-induced hypersensitivity reactions, including the clinical presentation, complications, management, and prognosis. Our systematic review of 30 ZNS-SCARs cases revealed key characteristics including a Japanese predominance (80%), a median latency of 23 days, and frequent hepatic involvement (26.7%), with human herpesvirus 6 reactivation confirmed in 66.7% of tested cases. Overall, 75% of patients improved following glucocorticoid-based therapies. This study aims to enhance awareness of SCARs associated with ZNS—including DRESS, SJS, and toxic epidermal necrolysis (TEN)—to support early recognition and timely clinical interventions.

## 1 Introduction

Zonisamide (ZNS) is a second-generation antiseizure medication (ASM) with multiple mechanisms of action, including blockade of voltage-gated sodium channels, inhibition of T-type calcium channels, enhancement of GABAergic inhibitory neurotransmission, and suppression of glutamate release (1). ZNS was first developed in 1974 and subsequently approved for clinical use in Japan (1989), the United States (2000), and Europe (2005), and was later introduced in China in 2009 (2). ZNS had become a widely used treatment option for epilepsy due to its broad-spectrum efficacy, favorable tolerability, minimal drug–drug interactions, and the lack of requirement for frequent therapeutic drug monitoring. Its relatively favorable safety profile during pregnancy and associated appetite suppression further supports its use in selected patient populations (3). Common adverse effects include somnolence, anorexia, headache, and dizziness; these are generally mild and often diminish or resolve with continued therapy (4).

Despite its overall safety, ZNS has been increasingly associated with rare but potentially life-threatening SCARs, most notably drug rash with DRESS, SJS, and TEN. These reactions can severely impact patients’ quality of life and impose substantial socioeconomic burdens. Here, we report the first documented case of ZNS-induced DRESS in China, which demonstrated potential progression to SJS, and provide a comprehensive review of the existing literature on ZNS-associated SCARs.

## 2 Case presentation

### 2.1 Patient background and initial presentation

A 37-year-old Chinese woman with a 2-year history of generalized tonic–clonic seizures (GTCS) presented for evaluation. She had no prior history of drug hypersensitivity or atopic disease. Her baseline antiepileptic regimen included levetiracetam 1,000 mg twice daily, which provided suboptimal seizure control with two to three breakthrough seizures annually. Due to inadequate control, ZNS, an antiseizure medication (ASM), was added with a titration schedule of 50 mg daily in the first week, increased to 100 mg daily in the second week, and maintained thereafter. On day 57 of combination therapy, she developed polymorphous erythematous lesions, presenting as small, pruritic, symmetrically distributed papules on the upper extremities. Over the next 3 days, the rash generalized, accompanied by severe pruritus and edema of the eyelids and facial skin. Initial outpatient treatment with methylprednisolone, vitamin C, and loratadine was ineffective. She was admitted to the dermatology department on hospital day 1 with persistent rash and edema.

### 2.2 Diagnostic workup and clinical course

**Figure 1:**
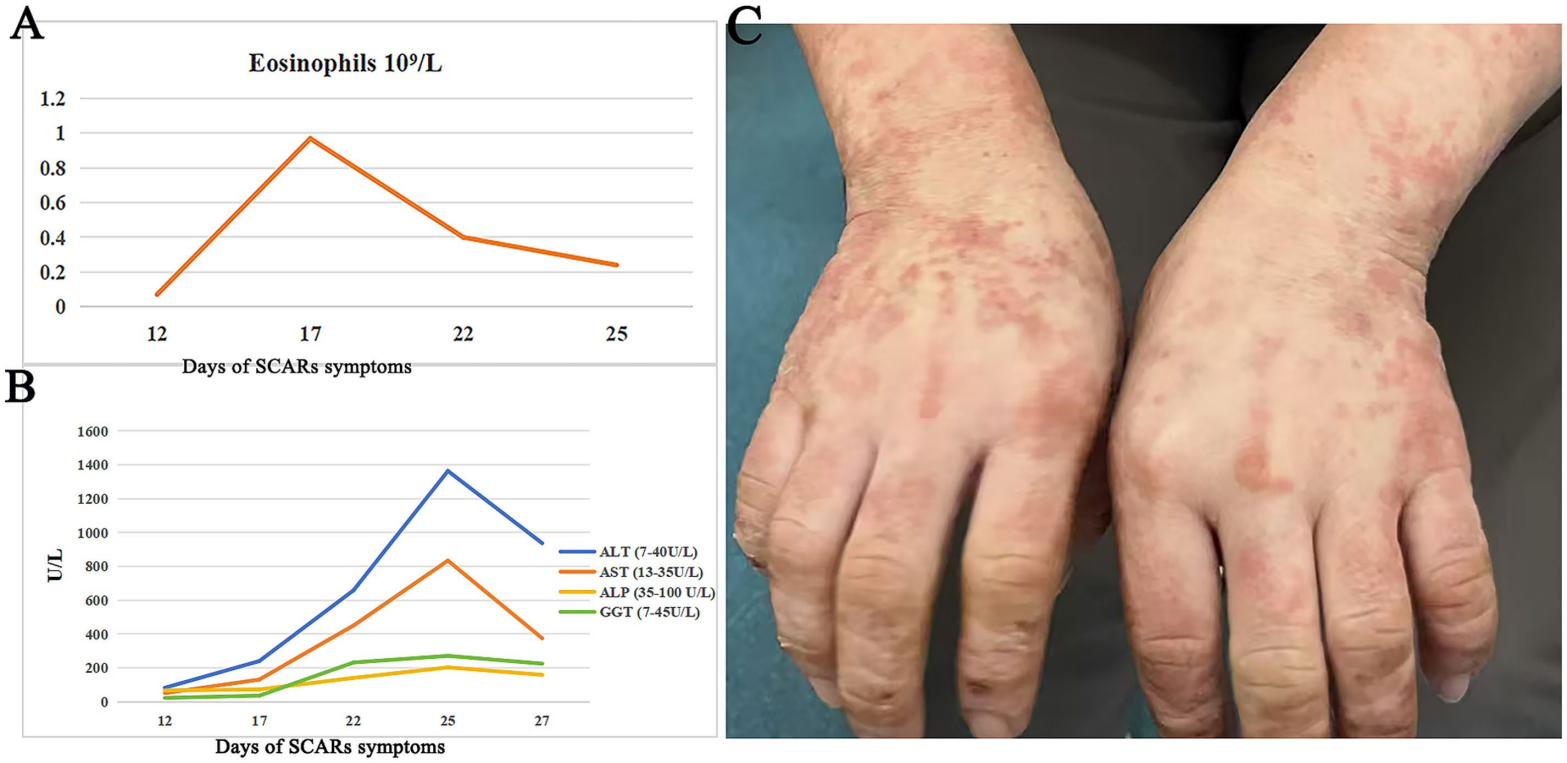
Changes in laboratory tests and rash manifestations during the disease course. **(A)** Changes in eosinophils count during the clinical course of SCARs. **(B)** Changes in alanine transaminase (ALT), alkaline phosphatase (ALP), and gamma-glutamyltransferase (GGT) during the clinical course of SCARs. **(C)** Erythematous macules, papules, and crusted lesions were visible on both hands after treatment.

On admission, vital signs were: temperature 37.1 °C, heart rate 113 bpm, respiratory rate 21/min, blood pressure 108/78 mmHg, and SpO₂ 95%. Physical examination revealed diffuse, symmetrical erythematous patches with scattered papules on the face, trunk, and limbs, accompanied by scales, scratch marks, erosion, exudation, crusting, and facial/eyelid swelling. Auspitz sign was negative. Laboratory evaluation showed elevated liver enzymes (ALT 83 U/L, AST 51 U/L) and mildly decreased albumin (37. 8 g/L); other serological and infectious workups were unremarkable. ZNS was withheld during the initial three hospital days; however, the patient self-administered ZNS on hospital day 4, resulting in rapid deterioration by hospital day 6, including worsening rash, purpura on the face, high fever (38. 8 °C), and progressive liver dysfunction with rising ALT, AST, and alkaline phosphatase, eosinophilia developed concurrently (Figures 1A,B). Using the Regi-SCAR scoring system, she scored five points, consistent with probable drug rash with DRESS (Table 1), with SJS not excluded. The patient declined a skin biopsy procedure due to personal concerns. ZNS was immediately discontinued upon patient disclosure of self-rechallenge.

**Table 1:** Evaluation of this case according to the RegiSCAR Diagnostic Criteria for DRESS.

Criteria	Score	Comments
Acute skin eruption		
More than 50% body surface area affected	Y = 1	
Rash characteristic of DRESS	Y = 1	Suggestive rash: ≥2 symptoms: purpuric lesions (other than legs), infiltration, facial edema, psoriasiform desquamation
Biopsy suggesting DRESS	U = 0	
Fever > 38.5°C	Y = 0	
Lymphadenopathy (>1 site, >1 cm)	U = 0	
Internal organ involvement	Y = 1	Score 1 for each organ involvement, maximal score:2
Eosinophilia		Maximum of 2 points
Eosinophilia ≥0.7×109/L or ≥10% if WBC < 4.0×109/L	Y = 1	
Eosinophils> ≥ 1.5×109/L or ≥20% if WBC < 4.0×109/L		Score 2 when ≥1.5 × 10′/L or ≥20% if WBC < 4.0 × 109/L
Atypical lymphocytes	U = 0	
Thrombocytopenia		
Additional parameters		
Resolution in>15 days	Y = 0	
Exclusion of: antinuclear antibodies, blood culture, serology for hepatitis A, B, and C, chlamydia, or mycoplasma	Y = 1	Score 1 if three tests of the following were performed and all were negative: hepatitis A virus, hepatitis B virus, mycoplasma, chlamydia, antinuclear antibody, blood culture
Total scores	5	Diagnosis requires three or more criteria. Likelihood of diagnosis based on total score: <2 = no; 2–3 = possible; score 4–5 = probable; score > 5 = definite.

DRESS, drug reaction with eosinophilia and systemic symptoms; RegiSCAR, Registry of Severe Cutaneous Adverse Reactions; U, unknown/detailed information unavailable; Y, symptom matches the description.

### 2.3 Treatment and outcome

The patient received intravenous prednisone acetate 60 mg daily with calcium gluconate supplementation. Intravenous calcium gluconate was administered short-term for its adjunctive antipruritic and membrane-stabilizing effects, and was discontinued once systemic corticosteroid therapy took full effect. After 3 days, partial rash improvement allowed reduction of prednisone to 40 mg daily, but worsening facial edema and rash-related pain prompted reinstatement of 60 mg daily. Spironolactone and topical clobetasol were added, along with intensified intravenous fluids and hepatoprotective therapy. Gradual improvement was observed, including decreased pain, partial desquamation, and recovery of liver function. The patient declined intravenous immunoglobulin (IVIG) therapy and self-discharged after 18 days of hospitalization. Ten days post-discharge, she demonstrated further clinical improvement, although residual erythematous patches, papules, and scabs persisted on the face and upper limbs (Figure 1C). For seizure management throughout hospitalization and after discharge, the previously administered levetiracetam was continued as monotherapy. The patient was advised to follow up in the neurology outpatient clinic for ongoing monitoring of seizure control.

## 3 Literature review

**Figure 2:**
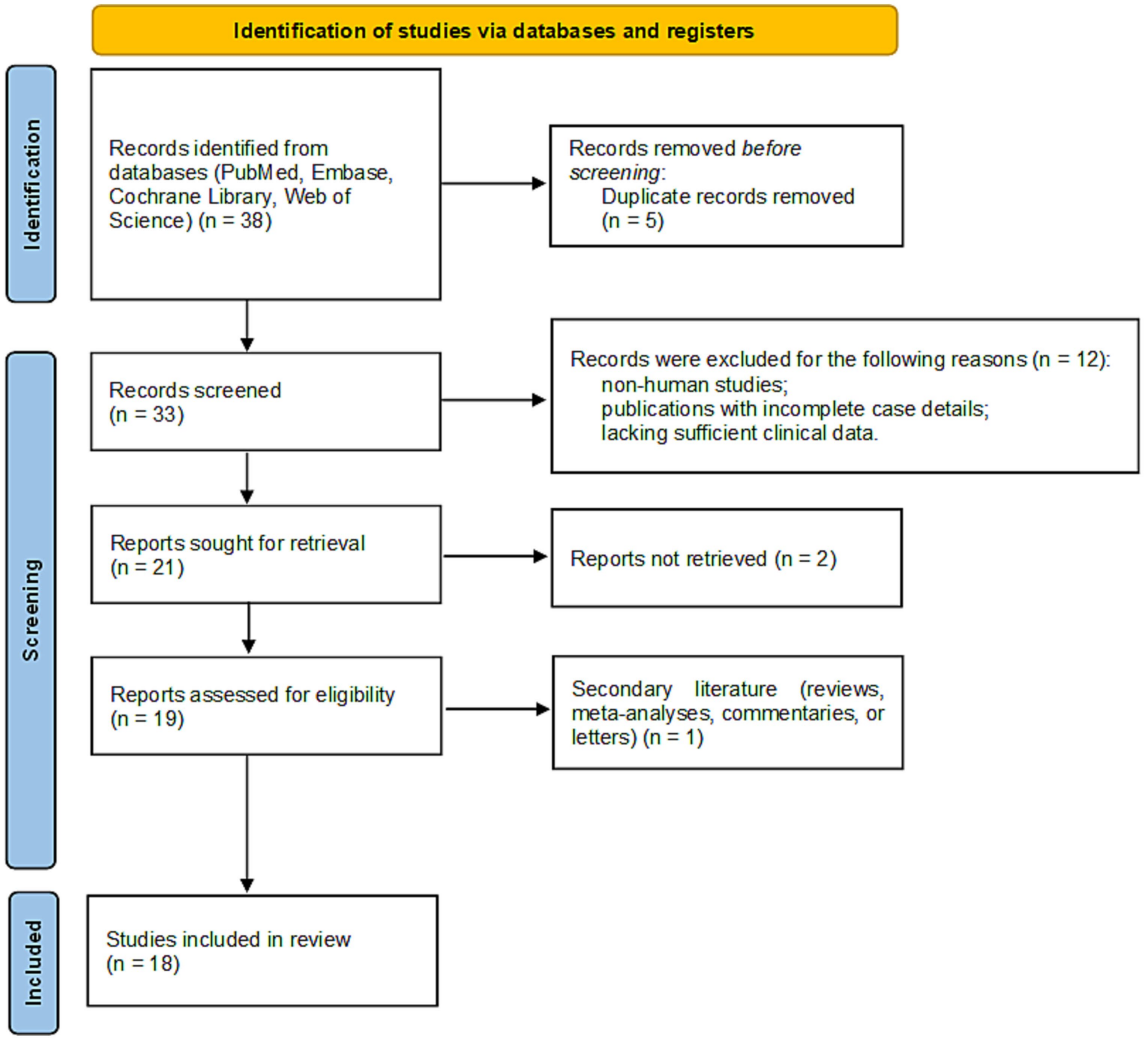
PRISMA flow diagram of the study selection process. This chart outlines the steps of database searching, screening, and eligibility assessment that resulted in the final number of studies included in the systematic review.

We conducted a systematic literature review in accordance with the Preferred Reporting Items for Systematic Reviews and Meta-Analyses (PRISMA) guidelines. The search was performed across four electronic databases: PubMed, Embase, Cochrane Library, and Web of Science. The following search terms were used, combining Medical Subject Headings (MeSH) and free-text keywords: (“Zonisamide” OR “ZNS”) AND (“DRESS” OR “Drug Reaction with Eosinophilia and Systemic Symptoms” OR “DIHS” OR “Stevens-Johnson Syndrome” OR “SJS” OR “Toxic Epidermal Necrolysis” OR “TEN” OR “severe cutaneous adverse reaction” OR “SCAR”). No language or date restrictions were applied, and the search covered all records from database inception through September 2025. Study eligibility was restricted to original case reports or case series documenting ZNS-associated SCARs, including DRESS, SJS, TEN, or their overlap syndromes. Exclusion criteria encompassed non-human studies, irrelevant topics, publications with incomplete case details or insufficient clinical data, and duplicate publications. The literature selection process is detailed in a PRISMA flow diagram (Figure 2). After excluding duplicates and studies with incomplete case details, 18 publications were included in the final analysis (5–22). Along with our reported case, a total of 30 cases were identified, representing four countries: Japan (*n* = 24), the United States (*n* = 2), African American (*n* = 1), and China (*n* = 1).

## 4 Results

(1) Case demographics and distribution

A total of 30 cases were analyzed, comprising 12 cases of SJS, nine cases of TEN, eight cases of drug rash with DRESS, and one case of overlap between SJS and TEN (SJS and TEN). The median age of patients was 41.5 years (range 2–78 years), and the male-to-female ratio was 18:11. The median latency period from ZNS exposure to the onset of symptoms was 23 days (range 10–593 days).

(2) Clinical features and organ involvement

Among the 30 cases, organ involvement was observed in 16 cases (53.3%, 16/30), with the liver being the most commonly affected organ (*n* = 8), followed by the lungs (*n* = 3), kidneys (*n* = 2), and digestive system (*n* = 3). In terms of viral reactivation, six cases (66.7%, 6/9) tested positive for human herpesvirus 6 (HHV-6).

(3) Lymphocyte transformation test (LTT) and pathology

The LTT was performed in seven cases (85.7%, 6/7), supporting an immune-mediated mechanism in these reactions. Pathological results were available for seven cases, contributing further to the clinical characterization of these adverse events.

(4) Treatment and clinical outcomes

Treatment varied depending on the severity of the reactions. Glucocorticoids (GC) were administered to seven patients, while combination therapies included GC with hemodialysis in one case, GC with IVIG in two cases, GC with IVIG and hemodialysis in one case, and IVIG monotherapy in three cases. Follow-up assessment showed clinical improvement in 12 of the 16 patients (75%). Of the 16 patients, four achieved complete recovery during hospitalization (25%), with treatment regimens including intermediate-dose GC (*n* = 1), pulse-dose GC with IVIG (*n* = 1), and IVIG monotherapy (*n* = 2). Detailed information can be found in Table 2.

**Table 2:** Literature review on previously reported ZNS-related SCARs.

Author, years	No.	Gender	Age (years)	Latent period	Race	ASMs	Types of skin lesions	Complication	Past history	Treatment of skin lesions	Follow-up ASMs	Prognosis	HHV-6	Pathology	LTT	Other viral infection
Shibuya et al. 2015 (10)	1	F	46	41d	Japanese	ZNS	DRESS	Eosinophilic pneumonia	SAH, HHT	Medium dose GC	NA	Improvement	+	+	+	NA
Murata et al. 2011 (16)	2	M	72	25d	Japanese	ZNS	DRESS	Severe liver damage	Craniotomy of intracranial aneurysm, HT, DM, allergic rhinitis, and conjunctivitis	Medium dose GC	NA	Cured	−	NA	NA	HHV-7
Fujita et al. 2010 (17)	3	M	29	48d	Japanese	ZNS	DRESS	Acute kidney injury	NA	Medium dose GC + HD	NA	Improvement	+	+	NA	NA
Kyo M et al. 2010 (18)	4	M	62	35d	Japanese	ZNS	DRESS	Liver damage	NA	Low dose GC	NA	Improvement	−	NA	58d+	MCV
Hirose et al. 2009(19)	5	M	17	30d	Japanese	ZNS-CBZ	DRESS-TEN	Liver damage	NA	Medium dose GC + IVIG	NA	Improvement	+	NA	51d+	NA
Trivedi et al. 2002 (5)	6	M	11	56d	Japanese	CLB, LEV, VPA, ZNS	DRESS	Liver damage, respiratory distress	CBZ related rash	Pulse dose GC + IVIG	CLB, LEV, VPA	Cured	NA	NA	NA	NA
Onuma et al. 2012 (14)	7	M	61	NA	Japanese	ZNS	DRESS	NA	Cerebral hemorrhage, convulsion	NA	NA	NA	+	NA	NA	MCV
Uhara et al. 2013(13)	8	F	62	22d	Japanese	ZNS	DRESS	Liver damage	Brain tumor	Topical GC	NA	Improvement	+	NA	−	NA
Khan et al. 2016(8)	9	F	8	593d	American	ZNS	DRESS	Liver damage	NA	High dose GC	NA	Improvement	NA	NA	NA	NA
Vivar et al. 2017 (7)	10	F	2	18d	African American	LEV-ZNS	SJS/TEN	NA	Extreme prematurity born at 25 weeks of gestational age	IVIG	LEV	Cured	−	+	NA	NA
Finkelstein et al. 2011 (15)	11	M	21	NA	NA	ZNS	SJS	NA	CBZ related rash	NA	NA	NA	NA	NA	NA	NA
Teraki et al. 2008 (20)	12	M	71	23d	Japanese	VPA-ZNS	TEN	Pneumonia with pulmonary atelectasis developed	Surgical treatment of a cerebral aneurysm	IVIG	NA	Improvement	+	+	22d+	NA
Majeres et al. 2004 (21)	13	F	16	14d	American	VPA-ZNS	TEN	NA	Bipolar disorder	NA	VPA	Improvement	NA	+	NA	NA
Nishimura et al. 2014 (11)	14	F	56	10d	Japanese	ZNS	TEN	Extensive intestinal involvement	Fibromyalgia	High dose GC + IVIG + HD	NA	Improvement	NA	+	NA	NA
Rizzo et al. 2015 (9)	15	M	11.2	14d	NA	ZNS, VPA	TEN	Acute kidney injury	NA	IVIG	NA	Cured	NA	NA	NA	NA
Ihara et al. 2019 (6)	16	M	50	14d	Japanese	LEV, LTG, ZNS	SJS	Esophageal and small intestinal injury	NA	Pulse dose GC	NA	Improvement	NA	+	+	NA
Yoshioka et al. 2022(22)	17	F	14	21d	Japanese	ZNS	SJS	CMVs gastritis, hepatic failure	NA	High dose GC + Immunoglobulin against CMV	NA	Improvement	NA	NA	+	MCV
Kaniwa et al. 2013(12)	18	F	71	21d	Japanese	ZNS	TEN	NA	NA	NA	NA	NA	NA	NA	NA	NA
	19	F	57	18d	Japanese	ZNS	SJS	NA	NA	NA	NA	NA	NA	NA	NA	NA
	20	M	25	20d	Japanese	ZNS	SJS	NA	NA	NA	NA	NA	NA	NA	NA	NA
	21	M	17	33d	Japanese	ZNS, CBZ	TEN	NA	NA	NA	NA	NA	NA	NA	NA	NA
	22	M	52	32d	Japanese	ZNS	TEN	NA	NA	NA	NA	NA	NA	NA	NA	NA
	23	M	78	22d	Japanese	ZNS	TEN	NA	NA	NA	NA	NA	NA	NA	NA	NA
	24	M	6	24d	Japanese	ZNS	SJS	NA	NA	NA	NA	NA	NA	NA	NA	NA
	25	F	31	40d	Japanese	PHT, ZNS	SJS	NA	NA	NA	NA	NA	NA	NA	NA	NA
	26	M	63	18d	Japanese	ZNS	SJS	NA	NA	NA	NA	NA	NA	NA	NA	NA
	27	F	30	18d	Japanese	ZNS	SJS	NA	NA	NA	NA	NA	NA	NA	NA	NA
	28	M	56	30d	Japanese	ZNS	SJS	NA	NA	NA	NA	NA	NA	NA	NA	NA
	29	M	59	28d	Japanese	ZNS	SJS	NA	NA	NA	NA	NA	NA	NA	NA	NA
This study	30	F	37	57d	Chinese	LEV, ZNS	SJS	Hepatic failure	−	Medium-dose GC	NA	Improvement	NA	NA	NA	NA

ASMs, anti-seizure medications; CLB, clobazam; CMV, cytomegalovirus; DM, diabetes mellitus; F, female; GC, glucocorticoid; HD, hemodialysis; HHT, hereditary hemorrhagic telangiectasia; HT, hypertension; IVIG, intravenous immunoglobulin; LEV, levetiracetam; LTG, lamotrigine; LTT, the lymphocyte transformation test; M, male; NA, not available; SAH, subarachnoid hemorrhage; SJS, Stevens–Johnson syndrome; TEN, toxic epidermal necrolysis; VPA, valproate; y, years; ZNS, zonisamide.

58d+, 51d+, 22d+, positive LTT reactions obtained at 58, 51, and 22 days after onset of the disease.

## 5 Discussion

Hypersensitivity reactions (HSRs) are common idiosyncratic adverse drug reactions associated with the use of ASM. Importantly, these reactions are not always dose-dependent and can occur even after prolonged use, often without any clear temporal relationship to the initiation of the medication. Previous studies indicate that, despite ZNS’s generally favorable safety and tolerability profile, the incidence of ZNS-induced HSRs can reach up to 2% (23). Among these, SCARs such as drug rash with DRESS, SJS, and TEN are particularly concerning due to their potential lethality.

DRESS is a rare but potentially life-threatening systemic HSR, characterized by extensive skin eruptions, organ involvement, lymphadenopathy, eosinophilia, and atypical lymphocytosis (24). Its pathogenesis is multifactorial, involving drug-induced metabolic alterations, physicochemical properties, genetic predisposition, and viral reactivation. Eosinophilic infiltration in the skin contributes to tissue damage and systemic manifestations, highlighting the critical importance of early recognition and intervention (25). However, SJS and TEN represent severe mucocutaneous reactions, distinguished by widespread epidermal detachment, blistering, and multi-organ involvement. Over 90% of cases present with mucosal lesions affecting at least two distinct anatomical sites (26). These conditions exist along a spectrum: SJS involves <10% of body surface area (BSA), while TEN affects >30% BSA. Both are primarily mediated by drug-specific cytotoxic T lymphocytes (CTLs). Activation of CTLs is initiated through presentation of the offending drug by major histocompatibility complex (MHC) class I molecules on keratinocytes to CD8 + T cells, triggering T cell proliferation and apoptosis of epidermal keratinocytes, ultimately leading to extensive skin necrosis and detachment (27).

In our case, the patient initially presented with fever, facial edema, organ involvement, and a morbilliform rash. A Regi-SCAR score of 5 indicated probable DRESS. During disease progression, the patient developed a painful rash with epidermal detachment (<10% BSA), crusting, and purpura, particularly on the face. According to Bastuji-Garin’s SJS criteria, these features suggest progression from DRESS to SJS (28). This clinical course aligns with prior reports, including a case reported by Hirose et al., demonstrating a transition from DRESS to TEN (19). It was noted that our case developed a severe HSR following the concurrent use of levetiracetam (LEV) and ZNS. Previous studies have indicated that the combination of ZNS with other ASMs or prior administration of ASMs before ZNS may result in immunological cross-reactivity in some individuals, with an incidence rate ranging from 40 to 80%, though the precise mechanisms remain unclear (7). Some reports have also suggested that the prior use of LEV or valproic acid (VPA) could potentially trigger severe skin reactions, including TEN, but the underlying immunological mechanisms remain unclear (29). However, opposing viewpoints exist in the literature, whereas other studies question a direct causal relationship (23). Consequently, further investigation is essential to definitively determine whether the severe cutaneous adverse reaction in this case is attributable to the combination of ZNS and LEV. These observations underscore the importance of prompt drug discontinuation once a drug-induced rash is suspected. In this case, delayed recognition due to incomplete medication history and inadvertent self-administration likely exacerbated disease severity. It was clarified that the rechallenge provided near-definitive evidence of ZNS pathogenicity, highlighting the critical importance of immediate and complete drug withdrawal in cases of suspected severe adverse drug reactions, as well as the necessity for healthcare professionals to ensure patients understand this. It is recommended to strengthen medication management and education for inpatients to prevent similar incidents.

Notably, several risk factors for ASM-related HSRs have been identified, including female sex, aromatic ASM structures, and polytherapy (30, 31). Our case aligns with these risk factors; however, literature review indicates a higher proportion of male patients in ZNS-SCARs (18 males vs. 11 females), warranting further investigation. Allergy history is also critical. ZNS shares chemical similarity with sulfonamides, suggesting avoidance in individuals with known sulfonamide allergies (21). In our review, we found one case of TEN where the patient had no personal history of allergies, but the mother had a sulfonamide allergy, implying a potential genetic predisposition to allergic reactions to ZNS (21). Another study suggested that individuals with a history of penicillin allergies are more likely to develop allergic reactions to non-antibiotic sulfonamides, further underscoring the importance of avoiding ZNS in such patients (32). Consequently, it is not only essential to obtain a thorough allergy history from the patient, but also to consider the allergy profiles of family members, as they may offer additional valuable predictive insights.

The interplay between drug-induced reactions and viral infections is complex. In DRESS, 41% (70/172) of cases tested positive for human herpesvirus 6 (HHV-6), with viral reactivation in 74% (52/70) of these cases (24). Two cases in our review were HHV-6 negative, with one positive for HHV-7 (33). Both HHV-6 and HHV-7 reside in circulating CD4 + T cells, and HHV-7 reactivation may facilitate HHV-6 reactivation (34, 35). This highlights the importance of considering both HHV-6 and HHV-7 in the diagnosis of DRESS, particularly in cases where HHV-6 reactivation is absent. Moreover, our review identified two instances of TEN associated with HHV-6 activation, which suggests that HHV-6 infection is not exclusive to DRESS (19, 20). However, HHV-6 reactivation in DRESS may correlate with disease severity and prognosis, research indicates that DRESS cases with concurrent HHV-6 infection often present with more severe clinical features and a poorer prognosis (36). Furthermore, concurrent cytomegalovirus (CMV) infection was observed in three DRESS cases, potentially exacerbating clinical outcomes (11, 14, 18). CMV infection may exacerbate the clinical presentation and contribute to complications, with targeted IVIG therapy showing efficacy (18, 22). Unfortunately, CMV testing was not conducted in our case, and no HHV testing was performed, preventing confirmation of any viral infections. This highlights the need for further research to explore potential correlations between prolonged illness and undetected viral infections.

Histopathology remains pivotal for diagnosing DRESS, SJS, and TEN. DRESS typically shows spongiosis, acanthosis, vesiculation, dermal lymphocytic infiltration (perivascular), eosinophils, atypical lymphocytes, and occasional granulomas (37), and SJS/TEN is characterized by keratinocyte apoptosis progressing to widespread epidermal necrosis (27). In this case, diagnosis relied on clinical assessment due to absence of histopathology. Globally, only seven ZNS-induced SCAR cases included pathological confirmation. The LTT is another valuable tool for diagnosing drug allergies, as it detects drug-specific T-cell sensitization by measuring the proliferative response of peripheral blood mononuclear cells to suspected medications. To achieve optimal test sensitivity, LTT should be performed within 1 week after the onset of skin rashes in patients diagnosed with SJS or TEN and 5–8 weeks after the onset of symptoms in patients with drug reaction with DRESS, in order to achieve optimal test sensitivity and specificity (38). In our study, LTT positivity was 85.7% (6/7) for ZNS-induced HSRs. Genetic predisposition is also meaningful for diagnosing drug allergies. A study exploring genomic biomarkers associated with ZNS-related SCARs identified a strong correlation between Human Leukocyte Antigen (HLA) genes and the susceptibility to these reactions (12). Specifically, the *HLA-A02:07* allele was found to be a potential biomarker for Japanese individuals at risk of ZNS-induced SJS and TEN (12) Additionally, the alleles *HLA-A02:07, HLA-B46:01, HLA-B58:01,* and *HLA-DRB108:03* were shown to be mutually associated with SJS/TEN induced by other drugs such as allopurinol, lamotrigine, and carbamazepine. These findings suggest that shared *HLA* risk alleles may underlie a common immunogenetic mechanism contributing to drug-induced SJS/TEN across different agents. Notably, recent evidence demonstrates substantial population-level variation in *HLA* allele frequencies. For example, the prevalence of *HLA-A02:07* ranges from 1 to 22.7% across Chinese populations (typically 3–13% in most Han groups, but up to 22.7% in certain ethnic minorities from Guizhou Province), approximately 4.1% in Koreans, and 0.7–4.0% in Japanese cohorts. Likewise, *HLA-B46:01* shows a distinct regional pattern, being relatively common in southern China and Southeast Asia (5–17%) (39, 40) These marked interethnic and geographic differences in *HLA* allele distributions may critically influence genetic susceptibility to drug-induced hypersensitivity, further population-based studies are needed to validate these associations. Our study showed that two cases of drug-induced rashes linked to carbamazepine (CBZ)-a medication known to cause hypersensitivity reactions (5, 15). Given this, it may be crucial to investigate potential shared genetic susceptibility between ZNS and CBZ, particularly with respect to their respective HLA associations. However, in the Chinese population, no overlap in pathogenic genetic markers has been observed between ZNS and CBZ. Specifically, ZNS does not appear to share the same genetic markers as *CBZ*, such as *HLA-B15:02* and *HLA-A31:01* (41). This absence of shared markers in the context of ZNS-SCARs and CBZ hypersensitivity underscores the complexity of genetic susceptibility in drug reactions. As the discovery of novel genetic factors associated with antiepileptic drug-induced SCARs continues to evolve, it is conceivable that future studies may uncover a potential genetic linkage between these two drugs.

In the management of ZNS-SCARs, GC therapy remains the predominant treatment approach, as evidenced by the literature review, where 78.6% (11/14) of cases utilized GC therapy. However, variations in the initial dosing and administration protocols were observed across the cases. Our analysis indicates that treatment strategies are individualized, ranging from low-dose to pulse corticosteroid therapy, often combined with adjunctive treatments like IVIG for severe cases. Among the 14 reported cases with documented treatment regimens, 13 demonstrated effective therapeutic responses and favorable outcomes during hospitalization. Notably, one case, in which a low dose of GC was administered, experienced exacerbation of the rash and associated symptoms, highlighting the potential risks associated with suboptimal corticosteroid dosing (18). However, a significant limitation of the current literature is the lack of standardized, quantifiable dosing regimens (e.g., mg/kg/day) and well-documented treatment durations, which precludes a definitive analysis of dose–response relationships or optimal therapy length. In terms of clinical outcomes, four cases achieved full recovery during hospitalization. This included one case treated with an intermediate-dose of GC, two cases managed exclusively with IVIG, and one case that received a pulse dose of GC in combination with IVIG. These findings underscore the importance of individualized treatment strategies, as both GC and IVIG have demonstrated efficacy in the management of ZNS-SCARs. Specifically, for conditions such as drug reaction with DRESS, SJS, and TEN, prompt and systematic corticosteroid administration is generally recommended. This approach has been shown to effectively arrest disease progression and improve clinical outcomes. Furthermore, the role of IVIG as an adjunctive treatment has gained recognition for its ability to reduce the required dose of GC, enhancing the overall therapeutic efficacy (37). However, more cautions were advised when using IVIG as a monotherapy, as emphasized by Joly et al. who noted the potential for severe adverse events, including infusion reactions, hypertension, hypotension, and pulmonary embolism (42). In our case, the patient’s symptoms initially improved after 3 days of standardized GC therapy but worsened on the fourth day, possibly due to re-exposure to ZNS. Upon increasing the GC dosage, the patient’s symptoms again showed improvement, suggesting that the overall treatment regimen was ultimately effective.

Currently, there is a lack of comprehensive data regarding the overall recovery time for ZNS-SCARs. However, the available literature suggests that the prognosis for DRESS, SJS, and TEN induced by ZNS is generally favorable. In the case reported herein, while the rash persisted beyond the hospital discharge, remaining present even 10 days post-discharge, this outcome may be influenced by factors such as individual susceptibility, potential re-exposure to ZNS, or a concurrent HHV-6/7 infection. Therefore, an ideal follow-up plan for ZNS-SCARs survivors is recommended to include the following components: dermatological evaluation for skin healing and sequelae; monitoring of liver and renal function for late complications; vigilance for autoimmune phenomena, particularly after DRESS; neurological management with alternative antiepileptic drugs to maintain seizure control; and reinforced patient education on lifelong ZNS avoidance and allergy documentation. This multidisciplinary approach is essential to optimize long-term outcomes.

In summary, this study represents a single-case report supplemented by a literature review, which inherently limits the scope and generalizability of the findings. The absence of a systematic review approach and the reliance on a single case underscore the need for further research. Future multicenter, large-scale cohort studies are warranted to validate the findings and provide a more comprehensive understanding of the treatment and prognosis of ZNS-SCARs.

## 6 Limitations

This study is limited by its single-case design, which constrains the generalizability of the findings. The absence of histopathological confirmation and incomplete viral testing (HHV-6/7, CMV) limits mechanistic insights into ZNS-induced SCARs. Furthermore, the literature review was based on retrospective case reports and may be subject to information bias and incomplete data reporting. Future multicenter, prospective studies with standardized diagnostic and treatment protocols are needed to validate these findings and clarify the genetic and immunological determinants of ZNS-SCARs.

## 7 Conclusion

ZNS-SCARs present as DRESS, SJS, or TEN, and are characterized by extensive dermatological manifestations and multi-organ involvement. In patients hospitalized with rash, a detailed evaluation of both past and current medications with potential sensitizing effects is essential, and any suspected agents should be promptly discontinued. LTT may serve as a valuable diagnostic tool for identifying the causative drug. Early, adequate, and systematic administration of GC and IVIG can effectively mitigate disease progression and improve clinical outcomes; however, IVIG monotherapy should be approached with caution. With timely and accurate intervention, the overall prognosis of ZNS-SCARs is generally favorable.

## Data Availability

The datasets presented in this article are not readily available because of ethical and privacy restrictions. Requests to access the datasets should be directed to the corresponding authors.
